# Fluid–structure interaction simulation of visceral perfusion and impact of different cannulation methods on aortic dissection

**DOI:** 10.1038/s41598-023-27855-2

**Published:** 2023-01-20

**Authors:** Gyu-Han Lee, Woon Heo, Youngjin Lee, Tae-Hoon Kim, Hyungkyu Huh, Suk-Won Song, Hojin Ha

**Affiliations:** 1 Department of Interdisciplinary Program in Biohealth-Machinery Convergence Engineering, Kangwon National University, Chuncheon, Republic of Korea; 2 Department of Smart Health Science and Technology, Kangwon National University, 1 Gangwondaehak-Gil, Chuncheon, 24341 Republic of Korea; 3 Department of Thoracic and Cardiovascular Surgery, Gangnam Severance Hospital, Yonsei University College of Medicine, 211 Eonju-Ro, Gangnam-Gu, Seoul, 06273 Republic of Korea; 4 Vascular Access Center, Lifeline Clinic, Busan, Republic of Korea; 5 Daegu-Gyeongbuk Medical Innovation Foundation, Medical Device Development Center, Daegu, Republic of Korea

**Keywords:** Computational biology and bioinformatics, Diseases, Medical research

## Abstract

Hemodynamics in aortic dissection (AD) is closely associated with the risk of aortic aneurysm, rupture, and malperfusion. Altered blood flow in patients with AD can lead to severe complications such as visceral malperfusion. In this study, we aimed to investigate the effect of cannulation flow on hemodynamics in AD using a fluid–structure interaction simulation. We developed a specific-idealized AD model that included an intimal tear in the descending thoracic aorta, a re-entry tear in the left iliac artery, and nine branches. Two different cannulation methods were tested: (1) axillary cannulation (AC) only through the brachiocephalic trunk and (2) combined axillary and femoral cannulation (AFC) through the brachiocephalic trunk and the right common iliac artery. AC was found to result in the development of a pressure difference between the true lumen and false lumen, owing to the difference in the flow rate through each lumen. This pressure difference collapsed the true lumen, disturbing blood flow to the celiac and superior mesenteric arteries. However, in AFC, the pressure levels between the two lumens were similar, and no collapse occurred. Moreover, the visceral flow was higher than that in AC. Lastly, the stiffness of the intimal flap affected the true lumen's collapse.

## Introduction

Aortic dissection (AD) is a life-threatening cardiovascular disease characterized by the tearing of the intimal layer within the aortic wall. This results in the subsequent formation of a secondary flow channel known as a false lumen. The true and false lumens are separated using a membrane known as the intimal flap. AD is classified into two groups according to the region in which the dissection is propagated. If the ascending aorta is involved, it is considered Stanford type A, and if the ascending aorta is not involved, it is considered Stanford type B. Mortality and morbidity vary significantly depending on the type of AD^1^.

Type A AD usually propagates near the heart and requires urgent surgery. If not treated, this type of AD has a mortality rate of 50% in 3 days and reaches 80% at the end of the second week^2^. In contrast, type B AD is less urgent and is usually treated with medication if there are no major complications. However, approximately 25% of type B cases have been reported to develop subsequent aneurysm dilatation or rupture^3^, with a mortality rate of 5% in complicated type B AD per 30 days and 2% in uncomplicated type B AD per 30 days^4^. Therefore, intervention is needed in cases of acute complicated type B AD, and thoracic endovascular aortic repair is frequently performed. When thoracic endovascular aortic repair cannot be performed in complicated type B AD, open heart surgery should be considered^5^. During surgery for AD, malperfusion occasionally occurs during cardiopulmonary bypass (CPB)^6^.

CPB is commonly used to repair type A AD^7–9^. Different cannulation strategies have been adopted, depending on the surgeon’s preferences, techniques, and patient’s anatomical characteristics. However, there has been controversy over the selection of the optimal cannulation strategy to ensure good organ perfusion and reduce the risk of malperfusion^9^. The optimal cannulation site remains controversial, and a general consensus has not been reached owing to insufficient data^7^. Different cannulation strategies with various advantages and disadvantages have been used because of the different anatomical characteristics and flow patterns inside the aorta during CPB^7,9^.

**Figure 1:**
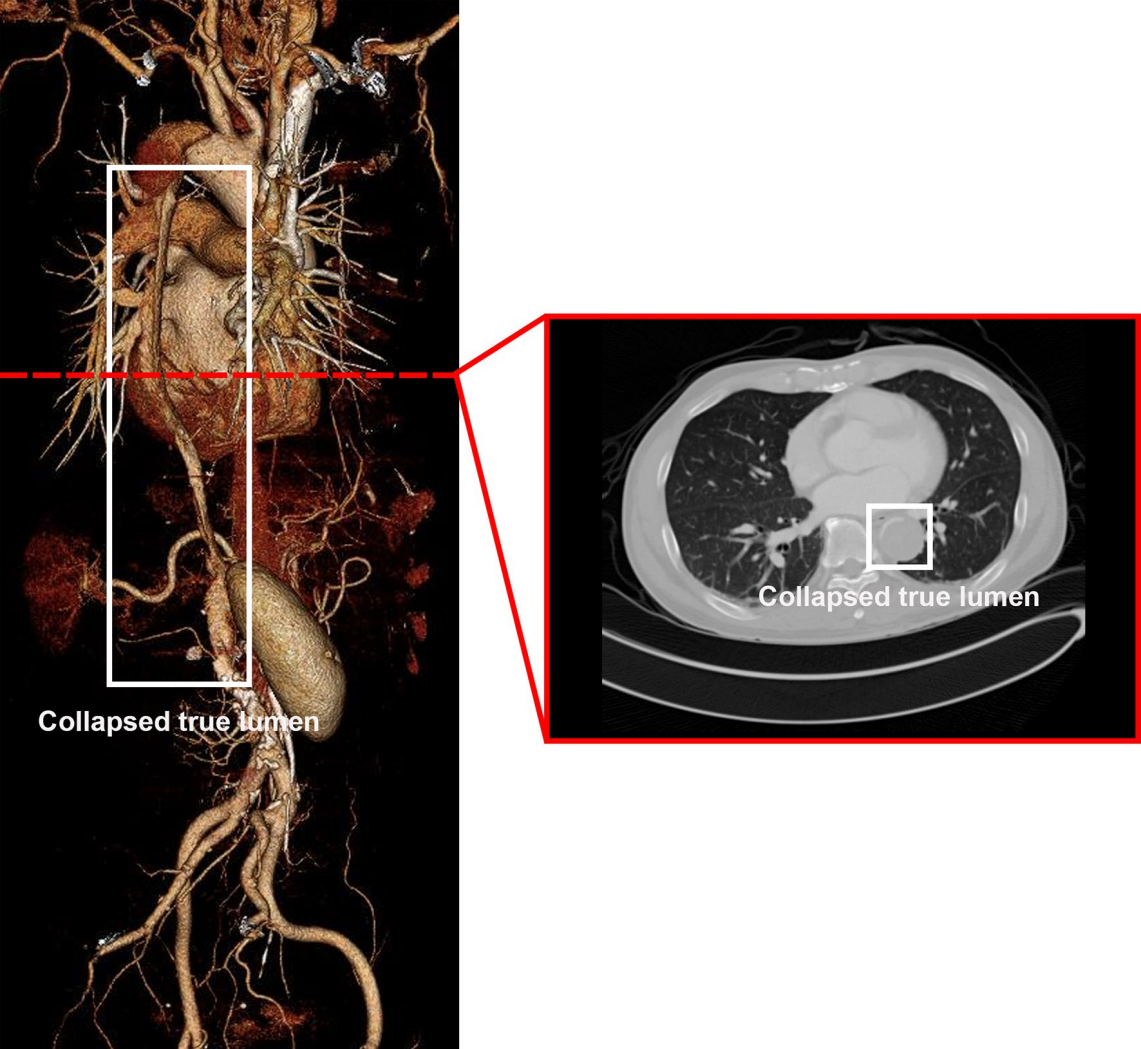
3D volume rendering and axial view of computed tomography in an AD patient with lower extremities malperfusion due to true lumen collapse. The image was reconstructed using TeraRecon (https://www.terarecon.com).

In AD, malperfusion is mostly caused by the collapse of the true lumen owing to the motion of the intimal flap, which obstructs branch vessels and results in end-organ ischemia (Fig. 1)^5,10^. Malperfusion can affect almost all major vascular beds, including the carotid, visceral, spinal, renal, and lower extremities, with varying frequencies and severity^5,10–12^. The malperfusion patterns depend on the region of the tear and dissection size. In the case of severe malperfusion, the affected organ may be injured by ischemia, which can significantly affect patient prognosis^11,12^. Thoracic endovascular aortic repair is a well-established method for treating malperfusion that comprises covering the primary entry tear, opening the true lumen, and thereafter relining the aorta to handle rupture. In addition, less invasive thoracic stenting has been used as an alternative to open repair, and end-organ malperfusion has been prevented by restoring visceral flow using endovascular fenestration^4^.

In the last decade, CFD has been widely used to investigate and understand the fluid-dynamic parameters and hemodynamic phenomena of AD with a high spatiotemporal resolution^13,14^. Previous CFD studies have revealed that hemodynamics play an important role in the progression of AD. For instance, the pressure difference between the true lumen and false lumen is a major factor in true lumen collapse and false lumen dilation^15^. High time-averaged wall shear stress (TAWSS) is associated with retrograde type A AD, tear initiation^16,17^, and false lumen evolution^18^. Low and oscillatory TAWSS are associated with shrinkage and thrombosis of the false lumen^15,17,18^. Therefore, although CFD is a powerful tool for AD studies, it has a limitation in that it generally assumes the intimal flap and aortic wall as rigid. To overcome this limitation, AD studies using fluid–structure interaction (FSI) simulations have recently been conducted with the development of computing power. The displacement of the vessel wall and its effect on the velocity and WSS around the true and false lumen was identified in FSI simulations^19^. Bäumler et al.^20^ compared FSI simulations by adjusting the intimal flap and aortic wall’s elastic modulus using four-dimensional flow magnetic resonance imaging (4D flow MRI). They found an elastic modulus with similar deformation to that of the patient and identified differences in hemodynamic phenomena according to the elastic modulus. In addition, FSI simulation, in vitro 4D flow MRI experiments, and catheter-based pressure measurement using patient-specific models provided valuable information on hemodynamic similarities and differences in AD^21^. Because the flow pattern resulting from complex geometry strongly affects AD, wall motion should be considered for better accuracy.

In our previous study^22^, we hypothesized that combined axillary and femoral cannulation (AFC) would recover the collapse of the true lumen of the dissected aorta and corresponding visceral malperfusion, which was confirmed via 4D flow MRI experiments using a specific-idealized in vitro model of AD. However, owing to the lack of spatial resolution of 4D flow MRI, the hemodynamic characteristics have not been investigated in detail. In particular, 4D flow MRI can only obtain low-resolution velocity information, limiting the investigation of other hemodynamic parameters such as pressure distribution.

The objective of this study was to perform FSI simulation of AD using different cannulation methods. The FSI results validate previous experimental results and provide fluid-dynamic parameters explaining the relationship between cannulation methods and visceral malperfusion in AD. This study presents three novel findings.

1. Intimal flap motion and corresponding hemodynamics in AD with different cannulation methods were investigated using FSI simulation. The pressure distribution on the intimal flap and the corresponding collapse of the true lumen were analyzed.
2. The effect of the cannulation method on visceral malperfusion in AD is presented.
3. The effect of the cannulation flow rate and stiffness of the intimal flap on the collapse of true lumen is investigated.

## Methods

**Figure 2:**
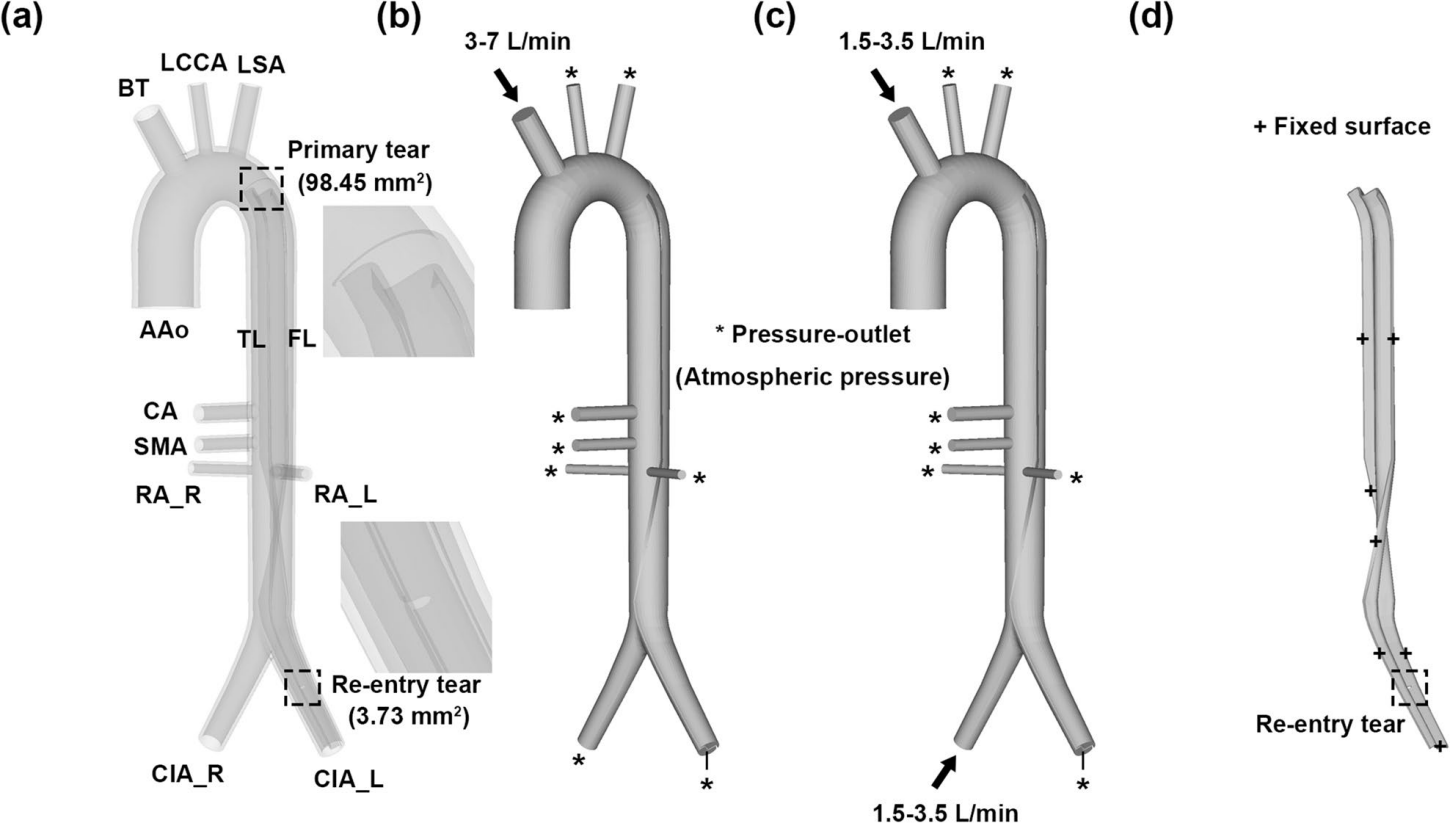
3D geometries and boundary conditions of AD. (**a**) Idealized model of a dissected aorta, (**b**) fluid domain of AC, (**c**) fluid domain of AFC, and (**d**) intimal flap. These images were made with Ensight (v.2021 R1, https://www.ansys.com).

Figure 2 provides an overview of the AD model and boundary conditions of the FSI simulation in this study. Our material properties and boundary conditions were set similar to those of the in vitro 4D flow MRI experiments of our previous study^22^, which are detailed in the following sections.

### Specific-idealized aortic dissection model

Visceral malperfusion is associated with a tear in the proximal descending thoracic aorta (DTA) in AD^23^. We created a specific-idealized model that showed visceral malperfusion in AD (Fig. 2a). The model, which comprised nine branches, was constructed using computer-aided design (CAD) software (SpaceClaim, v.2021 R1, ANSYS, Inc., PA, USA). The model had a large primary entry tear in the proximal DTA, which separated the true and false lumens, and a small re-entry tear in the left common iliac artery (CIA_L), allowing the flow in the false lumen to emerge in the true lumen. The area of the primary entry tear was 98.45 mm^2^, whereas that of the re-entry tear was 3.73 mm^2^. Additionally, the model had a uniform intimal flap with a thickness of 1 mm (Fig. 2d).

### 4D flow MRI experiment

In our previous study, we performed 4D flow MRI experiments using an in-vitro AD model^22^. The in-vitro model was constructed using an acrylic aortic wall and silicone intimal flap. The intimal flap was made of silicone rubber (Shore 20A; Trando 3D Medical Technology Co., Ltd., Ningbo, China). The working fluid was a mixture of water and glycerol in a 60:40 mass ratio, and all outlet arteries were opened to the reservoir, which was filled with working fluid to ensure the same outlet pressure. The 4D flow MRI was performed using a 3-T MRI machine (Skyra, Siemens AG, Munich, Germany). The 4D flow MRI sequence was acquired as follows for all experiments: scanner software version = VE11E, field of view = 176 × 352 × 56 mm^3^, matrix = 88 × 176 × 28, voxel size = 2 × 2 × 2 mm^3^, flip angle = 7°, coil = 16-channel coil. For axillary cannulation (AC), the following parameters were also considered: Venc = 100–200 cm/s, TE = 2.43–2.50 ms, TR = 10.18–11.10 ms, and one steady-state time frame. For AFC, the following parameters were used: Venc = 90–170 cm/s, TE = 2.43–2.50 ms, and TR = 5.16–5.63 ms. Furthermore, one steady-state time frame was used. In the cannulation experiments, the flow rate was maintained at a constant level with a centrifugal pump and controlled by opening and closing the valve. The flow rate was monitored using an electromagnetic flow meter (VN20; Wintech Process Co. Ltd., Gyeonggi-do, Korea). Two different cannulation conditions were used, AC and AFC. A steady flow rate of 3–7 L/min was applied to the brachiocephalic trunk (BT). AFC was performed at the same flow rate, but the total cannulation flow was divided in half into the BT and right common iliac artery (CIA_R).

### Computational fluid dynamics

The fluid domain was extracted from the CAD model (Fig. 2b,c). The fluid was modeled with a density and dynamic viscosity of 1060 kg/m^3^ and 0.0035 kg/m s (3.5 cP), respectively, similar to our 4D flow MRI experiment. A Newtonian fluid property was assumed because of the large diameters and high flow rates involved. As cannulation proceeded in the cardiac arrest state, the ascending aorta was treated as a wall. In the AC, the inlet condition was set to a uniform flow rate of 3–7 L/min applied to the BT, and the flow rate was halved in AFC and applied to the BT and CIA_R (Fig. 2b,c). All outlets were assumed to have the same constant pressure, as the pressure loss through the large aorta was negligible^24^. Rigid body and no-slip conditions were used for the aortic wall. A dynamic mesh function was used for deformation, and surfaces that made contact with the structural domains were set as fluid–solid interfaces. A fluid mesh of 1.6 million tetrahedral cells was selected based on the mesh independence test (Table S1). In this study, the maximum Reynolds number at the inlet was 4165, and the incompressible RANS equations were solved using the SIMPLE scheme to resolve pressure–velocity coupling. A shear stress transport k–ω turbulence model was used. A second-order upwind scheme was used for the momentum and turbulence equations. The convergence criterion was set as 0.001.

### Computational structural dynamics

The Young’s modulus of shore 20A can be estimated as approximately 0.732 MPa^25^, and its mechanical properties were applied in the Ogden 3rd order hyperelastic model using uniaxial, biaxial, and planar experimental data^26^ in this study. The strain-energy function for this model can be calculated as follows:

1 $$\psi =\frac{{\mu }_{1}}{{\alpha }_{1}}\left({\overline{{\lambda }_{1}}}^{{\alpha }_{1}}+{\overline{{\lambda }_{2}}}^{{\alpha }_{1}}+{\overline{{\lambda }_{3}}}^{{\alpha }_{1}}-3\right)+\frac{{\mu }_{2}}{{\alpha }_{2}}\left({\overline{{\lambda }_{1}}}^{{\alpha }_{2}}+{\overline{{\lambda }_{2}}}^{{\alpha }_{2}}+{\overline{{\lambda }_{3}}}^{{\alpha }_{2}}-3\right)+\frac{{\mu }_{3}}{{\alpha }_{3}}\left({\overline{{\lambda }_{1}}}^{{\alpha }_{3}}+{\overline{{\lambda }_{2}}}^{{\alpha }_{3}}+{\overline{{\lambda }_{3}}}^{{\alpha }_{3}}-3\right),$$

where ${\lambda }_{p}$ denotes the deviatoric principal stretches of the left-Cauchy–Green tensor, and ${\mu }_{p}$ and ${\alpha }_{p}$ are material constants. In this study, ${\mu }_{1}$ is − 2025.7 MPa, ${\alpha }_{1}$ is − 0.0706, ${\mu }_{2}$ is − 451.4 MPa, ${\alpha }_{2}$ is − 0.4271, ${\mu }_{3}$ is 1230.2 MPa, and ${\alpha }_{3}$ is − 0.2728. The compressibility terms are neglected assuming the incompressibility of the material.

For the hyperelastic material analysis, a solid mesh was used with approximately 55,000 20-noded hexahedral elements based on the mesh independence test (Table S2). Under the boundary condition, a fixed support was applied to all surfaces that did not make contact with the fluid domain and surfaces that made contact with the fluid domains were set as fluid–solid interfaces.

### Fluid–structure interaction

The FSI simulations were performed using a coupling system in the ANSYS workbench (v.2021 R1, ANSYS, Inc., USA) that connected ANSYS Fluent and ANSYS Mechanical software on a workstation (Dual Intel Xeon Gold 6148, 2.40 GHz CPU and 128 GB RAM). The ANSYS FSI simulation was based on a 2-way implicit iterative method in a transient coupled system. The forces or stresses on the fluid domain of the interface were converted to the solid domain, and the displacements on the solid domain of the interface were converted to the fluid domain in the coupling method. In this coupling, transferring information involves the calculation of weights and their subsequent use in data interpolation. The induced force on the intimal flap was obtained after the flow field was solved using ANSYS Fluent software. The displacement of the intimal flap was then solved using Ansys Mechanical software. This process was repeated until the end of the time step. The time step of the coupling system was set to 0.2 ms, and the end time was set to 2 s to stabilize the flap deformation. The minimum and maximum coupling iteration for each time step was set to 1 and 5, respectively. The maximum root-mean-square residuals for both the fluid and solid domains had to reach 0.01 to ensure convergence of the solution. The under relaxation factor was set to 1.0. To report the simulation results, we used the results of the end time step.

In this study, the continuity and momentum equations for an incompressible flow are as follows:

2 $$\nabla \cdot {{\varvec{v}}}_{f}=0,$$

3 $${\rho }_{f}\frac{\partial {{\varvec{v}}}_{f}}{\partial t}+{\rho }_{f}{{\varvec{v}}}_{f}\cdot \nabla {{\varvec{v}}}_{f}=-\nabla p+\nabla \cdot \mu \left(\nabla {{\varvec{v}}}_{f}+{\nabla }^{T}{{\varvec{v}}}_{f}\right),$$

where ${{\varvec{v}}}_{f}$ is the fluid velocity vector, ${\rho }_{f}$ is the fluid density, $p$ is the pressure, $\mu$ is the dynamic viscosity.

The momentum equation for structural domain is as follows:

4 $${\rho }_{s}\frac{\partial {{\varvec{v}}}_{s}}{\partial t}=\nabla \cdot {{\varvec{\sigma}}}_{s},$$

where ${{\varvec{v}}}_{s}$ denotes the solid velocity vector, ${\rho }_{s}$ the solid density, and ${{\varvec{\sigma}}}_{f}$ the solid stress tensor.

The boundary conditions at the FSI interface for the fluid and structural domains are given as

5 $${{\varvec{u}}}_{s}={{\varvec{u}}}_{f},$$

6 $${{\varvec{n}}}_{s}{{\varvec{\sigma}}}_{s}={{\varvec{n}}}_{f}{{\varvec{\sigma}}}_{f},$$

7 $$\frac{\partial {{\varvec{u}}}_{f}}{\partial t}={{\varvec{v}}}_{f},$$

where ${\varvec{u}}$ and ${\varvec{n}}$ are the displacement vector and normal vector, respectively, with the subscript $s$ indicating a property of the solid and $f$ of the fluid, ${{\varvec{n}}={\varvec{n}}}_{f}={-{\varvec{n}}}_{s}$ at the interface, and ${{\varvec{\sigma}}}_{f}$ is the fluid stress tensor.

To visualize and calculate data, Ensight (v.2021 R1, ANSYS, Inc., USA) and MATLAB (v.R2020a, The MathWorks, Inc., MA, USA) were used in this study.

### Effect of the intimal flap stiffness

Two additional simulations were performed to investigate the effect of the intimal flap stiffness. First, for AC at a flow rate of 7 L/min, CFD, in which all the bodies were rigid, was performed and its results were compared with those of the FSI simulation. Second, we applied more flexible mechanical properties than shore 20A for AC at a flow rate of 4 L/min using the neo-Hookean model. To validate that the results of the Ogden model and neo-Hooken model are similar, the Young's modulus of the previously estimated shore 20A (0.732 MPa) was applied to the neo-Hooken model, and the Poisson’s ratio was set to 0.49. To apply a more flexible material, the Young's modulus was set to 0.1 MPa and the Poisson's ratio was set to 0.49. The strain-energy function for this model can be calculated as follows:

8 $$\psi =\frac{\mu }{2}\left(\overline{{I }_{1}}-3\right)+\frac{1}{d}{\left(J-1\right)}^{2},$$

where $\overline{{I }_{1}}$ is the first deviatoric strain invariant, $J$ is the determinant of the deformation gradient, $\mu$ is the initial shear modulus of the material, and $d$ is the material incompressibility parameter. In this study, $\mu$ was 0.2456 MPa, and $d$ was 0.164/MPa for 0.732 MPa, and $\mu$ = 0.0336 MPa and $d$ is 1.2 MPa^-1^ for 0.1 MPa, respectively.

## Results

### Velocity fields and visceral flow

**Figure 3:**
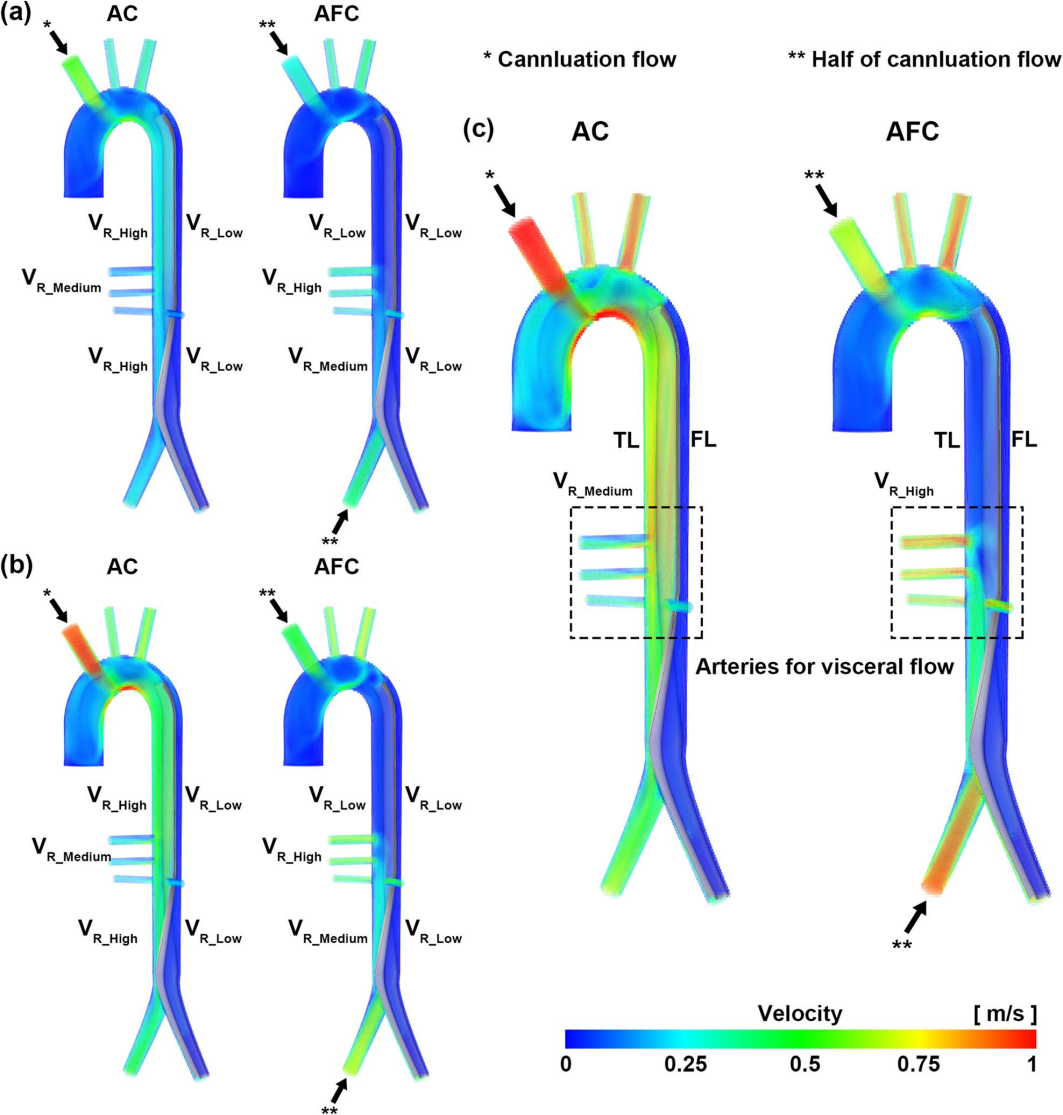
Velocity fields of AC and AFC. (**a**) 3 L/min, (**b**) 5 L/min, and (**c**) 7 L/min. These images were made with Ensight (v.2021 R1, https://www.ansys.com) using the FSI data.

**Figure 4:**
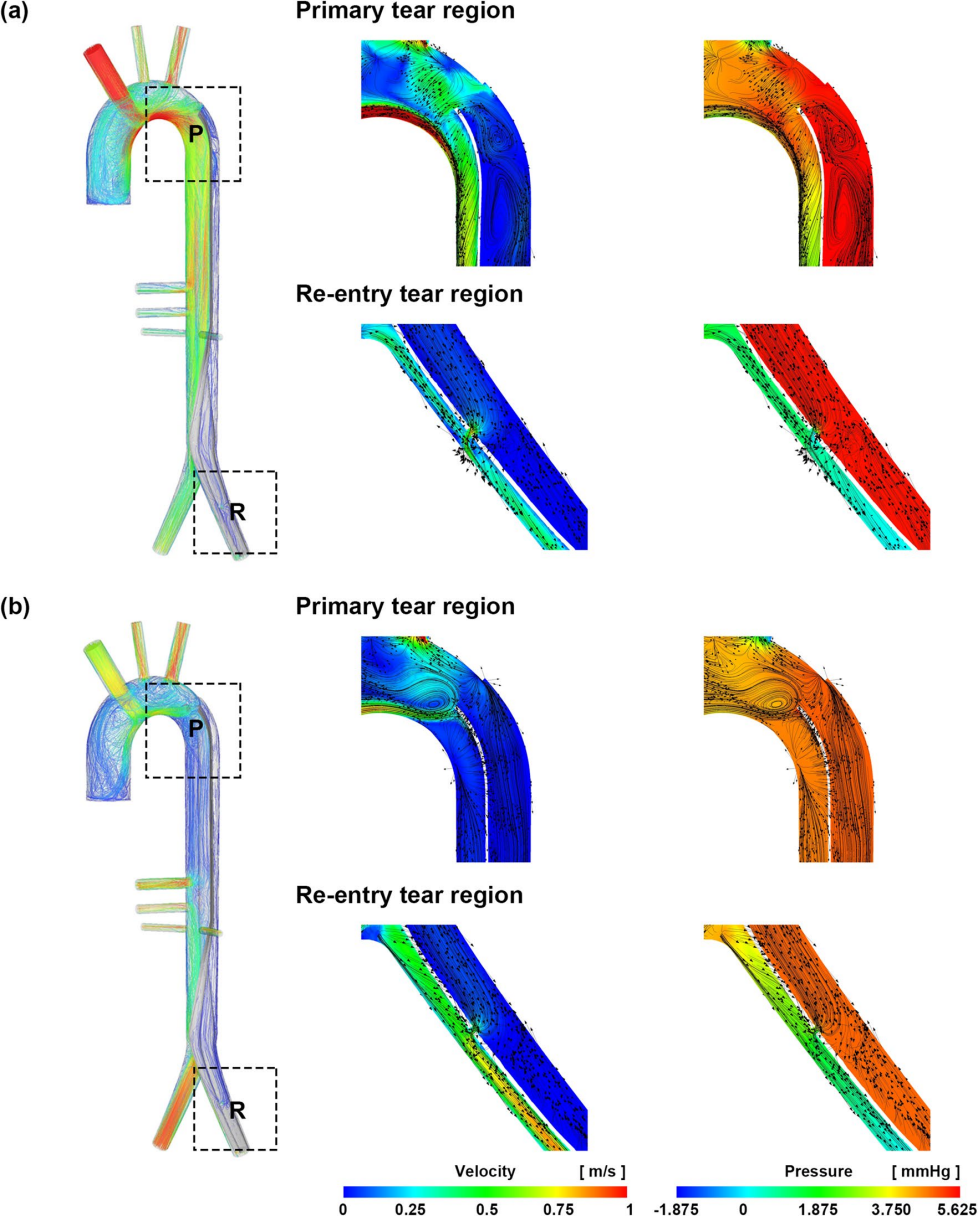
Characteristics of the tear regions for AC and AFC. (**a**) AC 7 L/min and (**b**) AFC 7 L/min. These images were made with Ensight (v.2021 R1, https://www.ansys.com) using the FSI data.

In the AC cases, the overall velocity in the true lumen was relatively higher than that in the false lumen, and recirculation was observed in the false lumen in the primary entry tear region of AC. In the re-entry tear region, a rapid flow was observed through the re-entry tear from the false lumen to the true lumen (Figs. 3, 4). In AFC, the velocity in the true lumen in the distal region was relatively higher, but the rate in the true lumen in the proximal region was low. In the celiac artery (CA), superior mesenteric artery (SMA), and renal arteries (RA_L and RA_R), AFC exhibited a higher velocity than AC in all cases (Fig. 3). In the primary tear region of AFC, recirculation was observed near the entrance of the true lumen; however, recirculation did not occur in the false lumen. In the re-entry tear region, AFC was observed to quickly exit from the false lumen to the true lumen through the re-entry tear, but the velocity in the true lumen was also higher than that of AC. Thus, the velocity at the exit of the common iliac artery was higher than that in the region of AC (Fig. 4).

The flow data of the FSI simulation for all arteries are summarized in Table 1. Visceral flow (through the CA, SMA, RA_L, and RA_R) increased when AFC was used instead of AC. As the flow rate in AFC increased from 3 L/min to 7 L/min, the amount of visceral flow increased from 96 to 132% compared with that in AC (Table 1). The flow data of the renal arteries were not included because of the spatial resolution of 4D flow MRI^22^. As the flow rate in the AFC increased from 3 to 7 L/min, the amount of visceral flow (CA and SMA) increased from 67 to 125% compared with that in AC (Table 2).

**Table 1:** Flow data of entire arteries in AC and AFC for the FSI simulation.

Cannulation	Arteries [L/min]								
	BT	LCCA	LSA	CA	SMA	RA_L	RA_R	CIA_L	CIA_R
AC at 3 L/min	3.000*	0.424	0.781	0.236	0.152	0.090	0.086	0.309	0.923
AC at 4 L/min	4.000*	0.553	1.030	0.309	0.204	0.118	0.122	0.421	1.261
AC at 5 L/min	5.000*	0.688	1.266	0.351	0.240	0.140	0.147	0.526	1.601
AC at 6 L/min	6.000*	0.875	1.600	0.392	0.279	0.167	0.172	0.573	1.862
AC at 7 L/min	7.000*	1.044	1.919	0.445	0.324	0.199	0.201	0.674	2.219
AFC at 3 L/min	1.500*	0.449	0.840	0.475	0.302	0.165	0.165	0.616	1.500*
AFC at 4 L/min	2.000*	0.594	1.112	0.640	0.406	0.226	0.227	0.804	2.000*
AFC at 5 L/min	2.500*	0.739	1.401	0.808	0.513	0.288	0.290	0.986	2.500*
ACF at 6 L/min	3.000*	0.891	16.651	0.979	0.620	0.353	0.354	1.161	3.000*
AFC at 7 L/min	3.500*	1.055	1.885	1.139	0.731	0.428	0.424	1.353	3.500*

*AC* axillary cannulation, *AFC* axillary and femoral cannulation, *BT* brachiocephalic trunk, *CA* celiac artery, *CIA_L* left common iliac artery, *CIA_R* right common iliac artery, *FSI* fluid–structure interaction, *LCCA* left common carotid artery, *LSA* left subclavian artery, *RA, L* left renal artery, *RA* right renal artery, *SMA* superior mesenteric artery.

*Indicates the inflow.

**Table 2:** Flow data of entire arteries in AC and AFC for the 4D flow MRI experiment^22^.

Cannulation	Arteries [L/min]						
	BT	LCCA	LSA	CA	SMA	CIA_L	CIA_R
AC at 3 L/min	2.99 ± 0.14*	0.38 ± 0.05	0.70 ± 0.06	0.25 ± 0.02	0.13 ± 0.01	0.17 ± 0.13	1.00 ± 0.08
AC at 4 L/min	4.01 ± 0.14*	0.51 ± 0.08	0.96 ± 0.08	0.38 ± 0.03	0.20 ± 0.02	0.23 ± 0.13	1.38 ± 0.14
AC at 5 L/min	5.06 ± 0.16*	0.69 ± 0.08	1.32 ± 0.12	0.62 ± 0.04	0.23 ± 0.04	0.14 ± 0.17	1.50 ± 0.12
AC at 6 L/min	6.02 ± 0.17*	0.89 ± 0.08	1.62 ± 0.10	0.81 ± 0.05	0.13 ± 0.02	0.18 ± 0.14	1.56 ± 0.05
AC at 7 L/min	7.06 ± 0.30*	1.03 ± 0.08	1.88 ± 0.12	1.02 ± 0.03	0.07 ± 0.02	0.42 ± 0.24	1.29 ± 0.16
AFC at 3 L/min	1.59 ± 0.05*	0.45 ± 0.05	0.83 ± 0.10	0.55 ± 0.03	0.31 ± 0.07	0.44 ± 0.12	0.65 ± 0.45*
AFC at 4 L/min	2.02 ± 0.07*	0.59 ± 0.04	1.08 ± 0.15	0.66 ± 0.05	0.44 ± 0.06	0.86 ± 0.17	1.45 ± 0.30*
AFC at 5 L/min	2.53 ± 0.07*	0.75 ± 0.04	1.37 ± 0.15	0.84 ± 0.04	0.58 ± 0.06	1.07 ± 0.18	2.07 ± 0.19*
ACF at 6 L/min	3.00 ± 0.07*	0.92 ± 0.08	1.67 ± 0.25	1.12 ± 0.04	0.76 ± 0.05	1.22 ± 0.30	3.04 ± 0.32*
AFC at 7 L/min	3.52 ± 0.13*	1.06 ± 0.10	1.95 ± 0.27	1.36 ± 0.05	0.83 ± 0.07	1.38 ± 0.27	3.42 ± 0.36*

*AC* axillary cannulation, *AFC* axillary and femoral cannulation, *BT* brachiocephalic trunk, *CA* celiac artery, *CIA_L* left common iliac artery, *CIA_R* right common iliac artery, *4D flow MRI* four-dimensional flow magnetic resonance imaging, *LCCA* left common carotid artery, *LSA* left subclavian artery, *SMA* superior mesenteric artery.

*Indicates that the inflow and flow data are expressed as the mean ± standard deviation.

### Pressure fields and intimal flap deformation

In this study, flap deformation was calculated using Eq. (9).

9 $${A}^{*}=\frac{{A}_{1}}{{A}_{0}}\times 100 \left[\mathrm{\%}\right],$$

where ${A}^{*}$ is the flap deformation, ${A}_{0}$ is the area of the true lumen before deformation (the initial area of the true lumen), and ${A}_{1}$ is the area of the true lumen after deformation.

**Figure 5:**
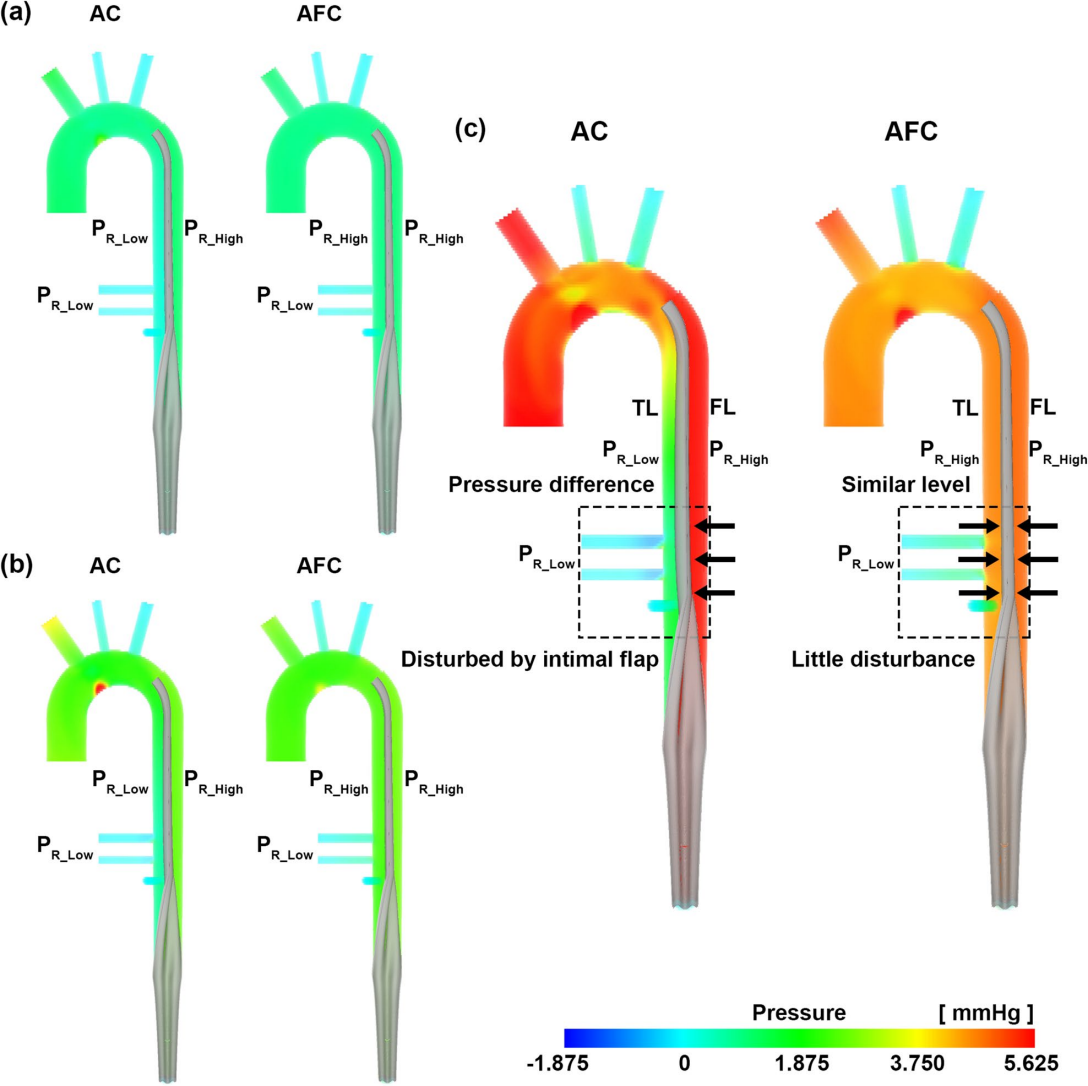
Pressure fields of AC and AFC. (**a**) 3 L/min, (**b**) 5 L/min, and (**c**) 7 L/min. These images were made with Ensight (v.2021 R1, https://www.ansys.com) using the FSI data.

**Figure 6:**
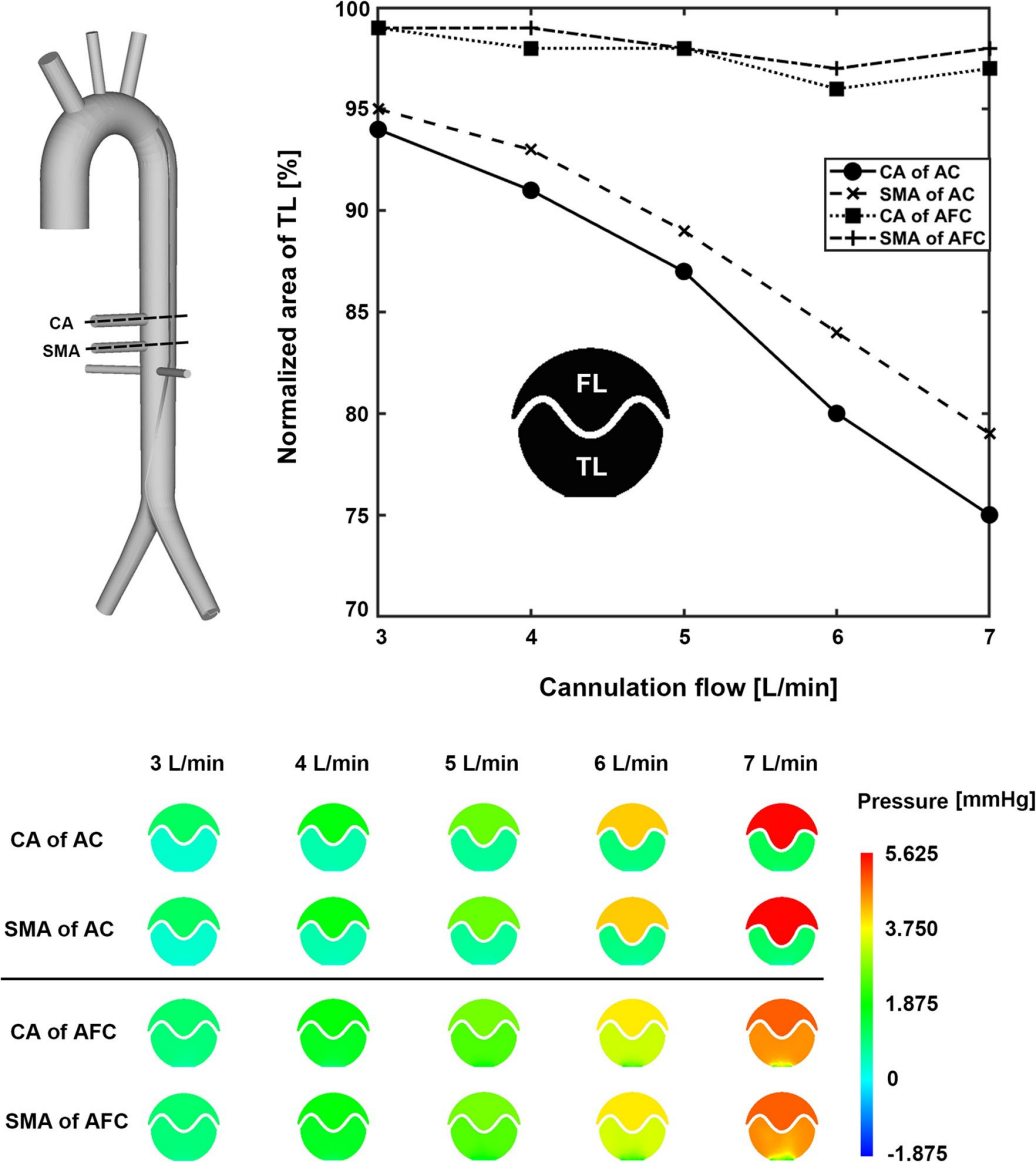
Normalized true lumen area of CA and SMA for AC and AFC. These images were made with Ensight (v.2021 R1, https://www.ansys.com) using the FSI data.

In the FSI simulation, as the AC flow rate increased, the pressure difference between the true lumen and false lumen increased. The pressure in the false lumen was relatively higher, and consequently, the flap collapsed toward the CA and SMA in the true lumen (Fig. 5). As the flow rate of AC increased from 3 to 7 L/min, the pressure difference between true lumen and false lumen at the CA increased from 0.82 to 4.50 mmHg, and that at the SMA increased from 0.80 to 4.26 mmHg (Table 3). The area of the true lumen at the CA decreased from 94 to 75%, and that at the SMA decreased from 95 to 79% (Fig. 6, Table 4). In AFC, the pressures in the true and false lumens were similar in all cases, and the intimal flap did not collapse toward the true lumen (Fig. 5). As the flow rate in AFC increased from 3 to 7 L/min, the pressure difference between true lumen and false lumen at the CA increased from 0.14 to 0.57 mmHg, and that at the SMA increased from 0.12 to 0.44 mmHg (Table 3). The area of the true lumen at the CA and SMA decreased from 98 to 97% (Fig. 6, Table 4).

**Table 3:** Pressure difference between the true and false lumens for AC and AFC in the FSI simulation.

Type	AC						AFC					
Region	CA			SMA			CA			SMA		
Flow	FL	TL	Diff	FL	TL	Diff	FL	TL	Diff	FL	TL	Diff
3 L/min	1.19	0.37	0.82	1.18	0.38	0.80	1.10	0.96	0.14	1.10	0.98	0.12
4 L/min	1.79	0.58	1.21	1.79	0.63	1.16	1.81	1.58	0.23	1.81	1.62	0.19
5 L/min	2.64	0.72	1.92	2.63	0.82	1.81	2.73	2.33	0.40	2.73	2.42	0.31
6 L/min	4.08	0.88	3.20	4.09	1.02	3.07	3.86	3.29	0.57	3.85	3.32	0.53
7 L/min	5.54	1.04	4.50	5.53	1.27	4.26	4.88	4.31	0.57	4.88	4.44	0.44

*AC* axillary cannulation, *AFC* axillary and femoral cannulation, *CA* celiac artery, *FL* false lumen, *FSI* fluid–structure interaction, *SMA* superior mesenteric artery, *TL* true lumen.

**Table 4:** Normalized area of the true lumen at the CA and SMA levels in AC and AFC^22^.

Type	AC				AFC			
Method	4D flow MRI		FSI		4D flow MRI		FSI	
Flow	CA [%]	SMA [%]	CA [%]	SMA [%]	CA [%]	SMA [%]	CA [%]	SMA [%]
3 L/min	87	75	94	95	99	99	99	99
4 L/min	80	74	91	93	100	100	98	99
5 L/min	75	72	87	89	100	101	98	98
6 L/min	73	72	80	84	102	103	96	97
7 L/min	72	71	75	79	103	104	97	98

*AC* axillary cannulation, *AFC* axillary and femoral cannulation, *CA* celiac artery, *FSI* fluid–structure interaction, *4D flow MRI* four-dimensional flow magnetic resonance imaging, *SMA* superior mesenteric artery.

In the 4D flow MRI experiment, as the flow rate of AC increased from 3 to 7 L/min, the area of the true lumen of the intimal flap at the CA level decreased from 87 to 72% of the area, respectively, and at the level of the SMA, from 75 to 71%. As the flow rate in the SMA increased from 3 to 7 L/min, the area of the true lumen of the intimal flap at the level of the CA changed from 99 to 103%, and at the level of the SMA, from 99 to 104% (Table 4).

### Intimal flap stiffness and deformation

**Figure 7:**
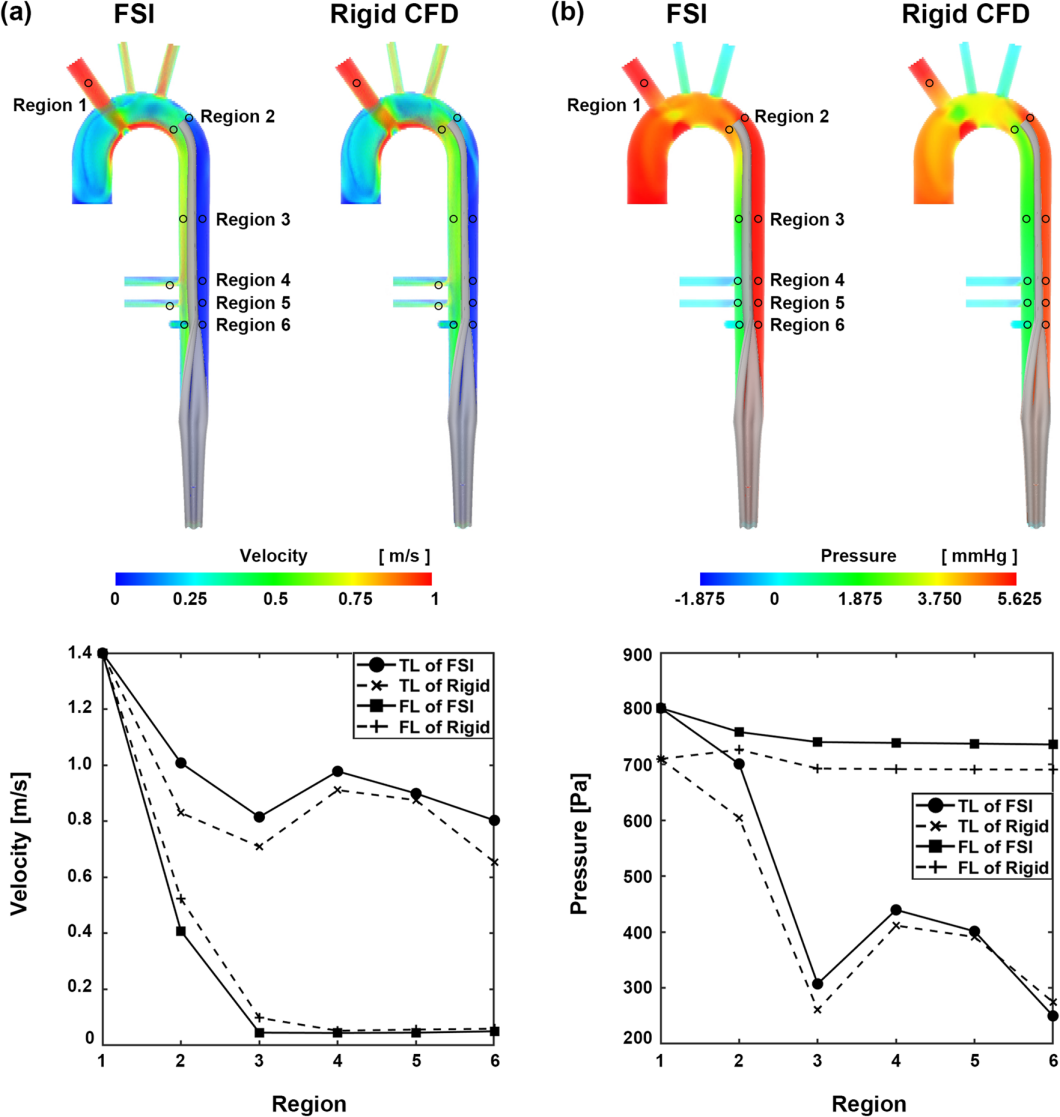
Velocity and pressure contour comparison of rigid CFD and FSI at an AC flow rate of 7 L/min. (**a**) Velocity and (**b**) pressure. The graphs show the maximum velocity and pressure in each region. These images were made from Ensight (v.2021 R1, https://www.ansys.com) using FSI data.

**Figure 8:**
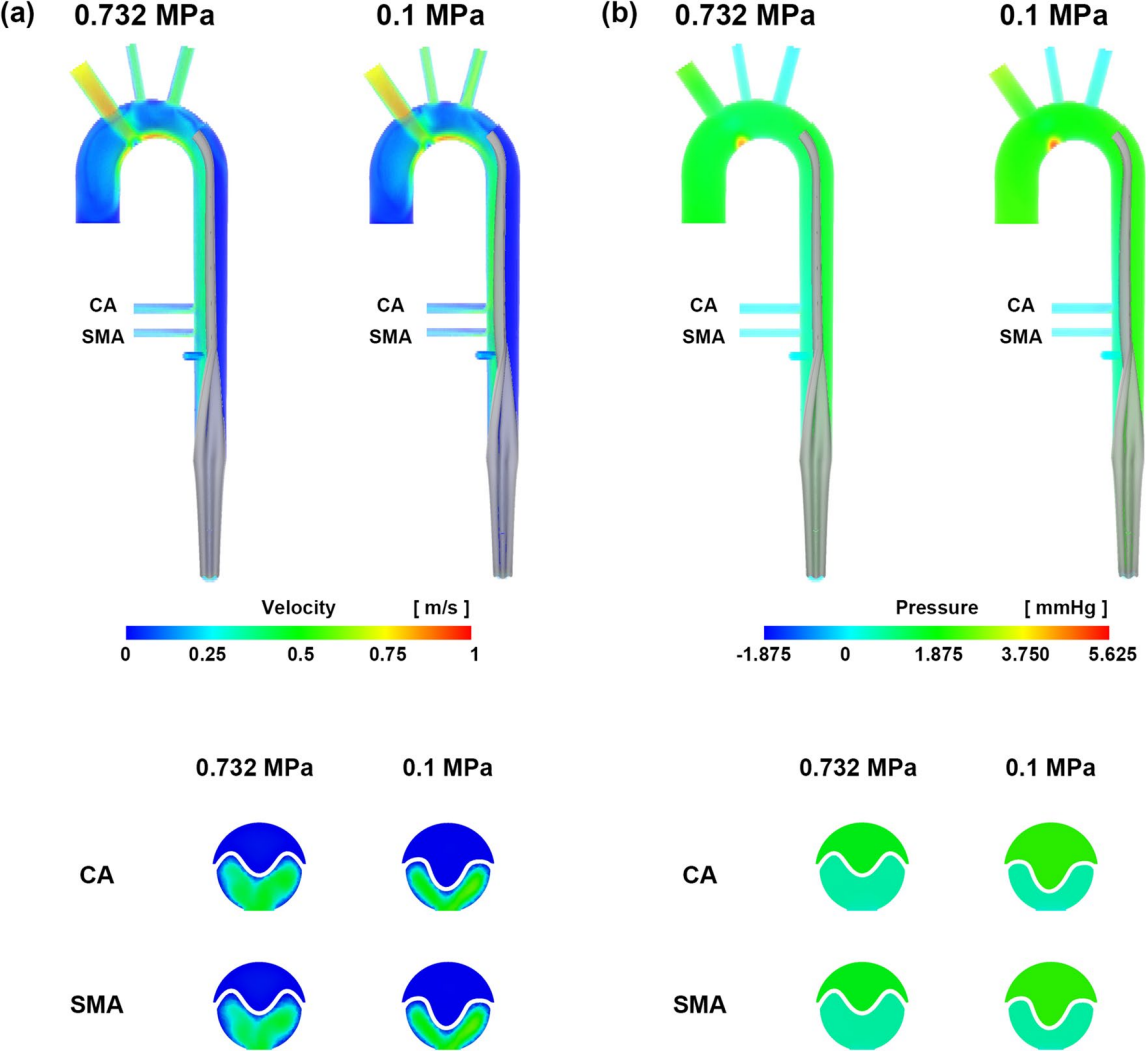
Velocity and pressure contour comparison according to stiffness at an AC flow rate of 4 L/min. (**a**) Velocity and (**b**) pressure. These images were made with Ensight (v.2021 R1, https://www.ansys.com) using FSI data.

Figure 7 compares the maximum velocity and pressure results in the corresponding regions of the CFD with a rigid intimal flap and FSI simulations for an AC flow rate of 7 L/min. In the FSI simulation, the maximum velocity in the true lumen was higher than that in the rigid CFD, whereas the maximum velocity in the false lumen was lower than that in the rigid CFD in all six regions. Except for region 6 in the false lumen, the maximum pressure was higher in both the true and false lumens than that in the rigid CFD. The collapse of the true lumen depends on the intimal flap stiffness. When the cannulation flow was 4 L/min, and the neo-Hookean model was applied, the flap deformation of CA was 95% and SMA was 96% at 0.732 MPa, respectively, and the CA was 67% and SMA was 71% at 0.1 MPa, respectively (Table 5). When more flexible mechanical properties were applied to the intimal flap, it was confirmed that even if the cannulation flow rate was only 4 L/min, additional collapse occurred (Fig. 8).

**Table 5:** Normalized area of the true lumen at the CA and SMA levels of AC and AFC for stiffness in the FSI simulation.

AC at 4 L/min		
Type	CA [%]	SMA [%]
3rd order Ogden (0.732 MPa)	91	93
Neo-Hookean (0.732 MPa)	95	96
Neo-Hookean (0.1 MPa)	67	71

*AC* axillary cannulation, *CA* celiac artery, *SMA* superior mesenteric artery.

## Discussion

The aim of this study was to investigate the hemodynamic parameters and phenomena associated with visceral malperfusion in AD by an FSI simulation. We hypothesized that AC could collapse the true lumen in the case of AD and cause visceral malperfusion owing to the pressure difference between the true lumen and false lumens. In our specific-idealized AD model, the major findings can be summarized as follows: (1) In AC, the intimal flap collapsed as the cannulation flow rate increased owing to the pressure difference between the true lumen and false lumen, but in AFC, there was no significant deformation because a similar pressure level existed between the true lumen and false lumen; (2) the amount of visceral flow was greater with AFC than with AC alone; (3) the stiffness of the intimal flap was an important factor in its deformation of the intimal flap; and (4) the FSI simulation exhibited a tendency similar to that of the 4D flow MRI experiment.

### Hemodynamics according to cannulation method

The pressure difference between the true lumen and false lumen is of interest because increased false lumen pressure may be associated with branch vessel obstruction^10^, and may vary depending on the size of the primary entry tear and re-entry tear, number of fenestrations between the true lumen and false lumen, and morphology of the branch vessel^27^. Chung et al.^28^ used an AD phantom to confirm that the collapse of the true lumen occurred sufficiently when the pressure in the false lumen exceeded the pressure in the true lumen by 1.1 mmHg or less. In AC, a pressure difference occurred between the true lumen and false lumen, with a minimum 0.82 mmHg at 3 L/min and maximum 4.5 mmHg at 7 L/min (Table 3). Consequently, the true lumen area decreased from 15 to 25%. However, in AFC, the pressure difference was a minimum of 0.14 mmHg at 3 L/min and a maximum of 0.57 mmHg at 7 L/min; thus, the area reduction was within 3% in all cases. Berguer et al.^29^ revealed that pressure equalization between the true lumen and false lumen is achieved only when the sum of the entire area in the AD phantom is at least 250 mm^2^. Our model had two tears, one in the primary entry tear in the DTA region and the other in CIA_L, and the sum of the areas was 101 mm^2^. We confirmed that the pressure difference was reduced using the AFC without generating fenestration in the intimal flap, resulting in no significant deformation of the intimal flap.

Malperfusion may cause myocardial, cerebral, visceral, spinal, and renal ischemia, resulting in various symptoms^5,10,30^. A large intimal tear in the proximal DTA causes malperfusion even in a pulsatile situation^22^. Our simulation results confirmed that the use of AC can cause visceral malperfusion and that the use of AFC increases visceral flow. The use of AFC increased from 97 to 133% compared to the use of AC alone, and the flow of other outlet branches also increased. Except for visceral flow, the flow of CIA_L increased from a minimum of 87% to a maximum of 103%. This proves that AFC has an advantage in delivering flow not only to the visceral but also to the lower extremities compared with AC. In addition, both AC and AFC exhibited low velocity in the false lumen, but only AC confirmed that the recirculation region occurred in the upper false lumen, which may better produce thrombosis^18^. The key message of this study is that the larger the pressure difference between the true lumen and false lumen in AC compared to AFC, the greater is the risk of collapse of the true lumen; AFC can resolve visceral malperfusion.

### Stiffness of the intimal flap

The threshold flow rate that causes true lumen collapse may vary depending on the intimal flap flexibility. Bäumler et al.^20^ confirmed that an FSI simulation was performed by changing the Young's modulus of the intimal flap from 0.8 to 0.02 MPa and that the displacement of the intimal flap increased from 1.4 to 13.4 mm. First, we compared rigid CFD and FSI at 7 L/min for AC, and the pattern of the velocity field was similar. However, in all areas shown in Fig. 6, the velocity in the true lumen of the FSI simulation was higher than that of the rigid CFD, and the velocity in the false lumen was lower than that of the rigid CFD. It was observed that the velocity in the true lumen increased and that in the false lumen decreased owing to the collapse of the true lumen. The pressure fields were also similar; however, except for region 6, the pressure of the FSI simulation was higher than that of the rigid CFD, indicating that resistance occurred owing to the deformation of the intimal flap at the same inlet flow rate, resulting in a high-pressure field. In actual clinical situations, a cannulation flow rate of ≥ 5 L/min is not used. Therefore, we performed FSI simulations at an AC flow rate of 4 L/min by applying 0.732 MPa, which similar to that in the existing model and 0.1 MPa lower than that of the neo-Hookean model. Comparing these two analyses, the area of the true lumen was reduced by 28% in the CA and 25% in the SMA at 0.1 MPa. This implies that the exact mechanical properties of the structural part are required for patient-specific FSI simulation and remain a limitation of the current FSI simulation^19,20^.

### Comparison with the 4D flow MRI experiment

In recent years, there has been an increasing number of studies comparing the results of FSI simulations directly with patient data acquired with 4D flow MRI^20^ or 4D flow MRI experimental data obtained using an in vitro model with an FSI simulation^21^. This study intended to confirm the potential of an FSI simulation by comparing it with a previously conducted 4D flow MRI experimental study and to investigate additional fluid-dynamic parameters and hemodynamic phenomena. A comprehensive comparison between the FSI simulation and 4D flow MRI showed similarities in the blood flow patterns obtained in the aortic region. Our objective was to demonstrate the potential for a consistent comparison of FSI simulations with 4D flow MRI experiments and direct application to other scenarios, given the morphological and pathological complexity of AD. The difference between the 4D flow MRI experiment and FSI simulation arises from the discrepancy between the models, inaccurate flap stiffness, and 4D flow MRI resolution. In our study, the differences between the FSI simulation and 4D flow MRI experiment were the amount and shape of the intimal flap in the AC. In a previous 4D flow MRI experiment^22^, as the cannulation flow increased, the area of the true lumen in AC decreased from 87 to 72% in the CA and from 75 to 71% in the SMA. In AFC, the area of the true lumen changed from 99 to 103% in the CA and from 99 to 104% in the SMA. Although there was a difference in deformation, similar results were obtained in that the intimal flap collapsed in AC, reducing the area of the true lumen, and there was almost no deformation in AFC. The amount of deformation is related to the stiffness of the intimal flap, as mentioned earlier. Our FSI used the mechanical properties of Shore 20A from the reference^26^. Due to the measurement uncertainty, the mechanical properties of the intimal flap applied to the FSI simulation may not be precisely identical with those in the 4D flow MRI experiment. In the 4D flow MRI experiments, the collapse occurred mainly in the SMA of the true lumen and, more significantly, in the CA in the FSI simulations. This seems to have occurred because the intimal flap extended into the entire area of the true lumen, unlike in the FSI simulation, to facilitate assembly with acrylic in the 4D flow MRI experiments. In addition, when producing multiple intimal flaps, some differences in thickness or mechanical properties exist between the intimal flaps owing to their complex geometries, and sensitive differences also occur in the process of combining the flaps with the acrylic model. In addition, the velocity field patterns and the increase in visceral flow exhibited good consistency. In the 4D flow MRI experiment, as the cannulation flow increased, the mean visceral flow in AC changed from 0.25 to 1.02 L/min at CA and from 0.13 to 0.07 L/min at SMA. In AFC, the mean visceral flow changed from 0.55 to 1.36 L/min at CA and from 0.31 to 0.83 L/min at SMA. There was also a difference in flow, but the visceral flow was higher in AFC than in AC. Thus, the potential of the FSI simulation was confirmed.

### Limitations

This study had several limitations. First, our model was specifically idealized, and the aortic wall was assumed to be rigid, except for the intimal flap. The rigid aortic wall may affect the distribution of hemodynamic parameters, but accurately capturing wall dynamics with a uniform aortic wall thickness is challenging^19^. The rigid aortic wall was set up under the same conditions as those in a previous study^22^. In the future, we will conduct a study considering the compliance of the aortic wall using a patient-specific model. Second, the fluid was assumed to be Newtonian. To consider non-Newtonian properties, several blood flow simulation studies^19,31–33^ have used the Carreau–Yasuda viscosity model. However, in blood vessels with large diameters, such as the aorta, Newtonian fluids are sufficiently acceptable^34^. Third, the cannulation flow rate does not exceed 5 L/min in clinical practice. Additional simulation cases confirmed that the low intimal flap stiffness at 4 L/min and clinical cannulation flow rate can cause visceral malperfusion. Fourth, we applied atmospheric pressure to all the outlet branches to ensure that they had the same pressure levels. This is a physiologically fatal limitation, and a pressure field different from that in clinical practice may occur and affect the deformation of the intimal flap. However, it was applied so that the pressure conditions in the simulation were the same as those in the previous 4D flow MRI experimental setup^22^. In a future study, physiological conditions will be applied using a three-element Windkessel model, as in other studies^19,20,34,35^. Finally, the zero-stress state was not considered. In practice, residual stress remains in the artery even in the unloaded state. This residual stress state depends on the thickness and composition of the artery, which should be considered to accurately predict the deformation.

## Conclusions

Using FSI simulation, we confirmed that when using AC, the pressure difference between the true lumen and false lumen allows the intimal flap to block blood flow to the CA and SMA in our specific-idealized AD model. As the cannulation flow rate increased in the AC, the intimal flap was further attached to the CA and SMA, indicating that the AC may induce visceral perfusion failure. When using AFC with added femoral cannulation, the pressure difference between the true lumen and false lumen in all cases was maintained at similar levels; thus, no significant deformation of the flap occurred and the amount of visceral perfusion was significantly higher than that of AC.

## Supplementary Information

Supplementary Tables.

## Data Availability

All data generated or analyzed during this study are included in this its Supplementary Information files.
