# Supplementary material for: Fluid–structure interaction simulation of visceral perfusion and impact of different cannulation methods on aortic dissection
Source: Sci Rep. 2023 Jan 20;13:1116. doi: 10.1038/s41598-023-27855-2 (PMC9860063; doi:10.1038/s41598-023-27855-2)
Supplement: Supplementary file 1 — Supplementary Tables. [file 41598_2023_27855_MOESM1_ESM.docx]

Fluid-structure interaction simulation of visceral perfusion and impact of different cannulation methods on aortic dissection

Gyu-Han Lee^1^, Woon Heo^3,4^, Youngjin Lee^2^, Tae-Hoon Kim^3^, Hyungkyu Huh^5^, Suk-Won Song^3,†^, and Hojin Ha^2,†^

^1^ Department of Interdisciplinary Program in Biohealth-Machinery Convergence Engineering, Kangwon National University, Chuncheon, Republic of Korea

^2^ Department of Smart Health Science and Technology, Kangwon National University, Chuncheon, Republic of Korea

^3^ Department of Thoracic and Cardiovascular Surgery, Gangnam Severance Hospital, Yonsei University College of Medicine, Seoul, Republic of Korea

^4^ Vascular Access Center, Lifeline Clinic, Busan, Republic of Korea

^5^ Daegu-Gyeongbuk Medical Innovation Foundation, Medical Device development Center, Daegu, Republic of Korea

^†^ Suk-Won Song and Hojin Ha are co-corresponding authors.

**Corresponding author**

Department of Thoracic and Cardiovascular Surgery, Gangnam Severance Hospital, Yonsei University College of Medicine, 211 Eonju-ro, Gangnam-gu, Seoul 06273, Republic of Korea. Tel: +82-2-2019-3384; e-mail: sevraphd@yuhs.ac (S.-W. Song).

Department of Smart Health Science and Technology, Kangwon National University, 1 Gangwondaehak-gil, Chuncheon 24341, Republic of Korea. Tel: +82-33-250-6310; e-mail: hojinha@kangwon.ac.kr (H. Ha);

**Table S1** Mesh independence test for fluid domain

| Type | AC 7 L/min | |
| --- | --- | --- |
| Flow | Elements number | Maximum velocity [m/s] |
| M1 | 1135309 | 1.55 |
| M2 | 1601193 | 1.59 |
| M3 | 3784296 | 1.61 |
| Difference [%] | M2/M1 | 2.58 |
|  | M3/M2 | 1.26 |

AC, axillary cannulation

Regarding the fluid domain, transient flow simulations were performed for different meshes to compare the results of maximum velocity. Solutions were considered mesh independent when the monitored parameters differed by less than 1.5% between two successively refined meshes. These results showed that M2 should be chosen for flow simulation.

**Table S2** Mesh independence test for solid domain

| Mesh | Elements number | Maximum displacement [mm] |
| --- | --- | --- |
| M1 | 3770 | 3.27 |
| M2 | 55496 | 3.67 |
| M3 | 224220 | 3.69 |
| Difference [%] | M2/M1 | 12.14 |
|  | M3/M2 | 0.51 |

Regarding the structural domain, static structural simulations were performed for different meshes to compare the results of maximum displacement. Solutions were considered mesh independent when the monitored parameters differed by less than 1% between two successively refined meshes. These results showed that M2 should be chosen for structural simulation.
